# Distal resection margins before and after fixation: a comparative analysis in rectal cancer surgery

**DOI:** 10.3389/fsurg.2026.1845405

**Published:** 2026-08-17

**Authors:** Akay Edizsoy, Orkhan Mirzayev, Hatice Barak, İbrahim Meteoğlu, Pars Tunçyürek

**Affiliations:** 1 Department of General Surgery/Surgical Oncology, Aydın Adnan Menderes University Faculty of Medicine, Aydın, Türkiye; 2 Department of General Surgery, Muğla Training and Research Hospital, Muğla, Türkiye; 3 Department of General Surgery, Aydın Adnan Menderes University Faculty of Medicine, Aydın, Türkiye; 4 Department of Pathology, Aydın Adnan Menderes University Faculty of Medicine, Aydın, Türkiye

**Keywords:** cancer, distal, fixation, margin, rectal, resection, shrinkage

## Abstract

**Background:**

Formalin fixation may cause measurable changes in the dimensions of surgical specimens, which may potentially affect the pathological assessment of distal margins in rectal cancer surgery. This study aimed to evaluate the differences between distal margin measurements obtained from fresh specimens and those recorded after fixation.

**Methods:**

Rectal cancer specimens from patients who underwent anterior or low anterior resection were retrospectively analyzed. Distal margins were measured macroscopically on fresh specimens during intraoperative pathological assessment and then remeasured after formalin fixation. Differences between the prefixation and postfixation measurements were calculated, and the shrinkage rates were determined.

**Results:**

Sixty patients were included in this analysis. The mean distal margin decreased from 4.39 ± 1.95 cm in fresh specimens to 3.90 ± 2.04 cm after fixation, corresponding to a mean reduction of 0.49 cm (95% CI: 0.26–0.71 cm) and an average shrinkage of 13.2%. No specimen had a distal margin shorter than 1 cm on fresh measurement; however, after fixation, two specimens were reported to have margins below this threshold. During a median follow-up of 46.5 months, exploratory analyses did not identify a clear association between the postfixation distal margin category (<2 cm vs. ≥ 2 cm) and oncological outcomes; however, these analyses were based on small subgroups and should be interpreted with caution. Among the two patients with a distal margin <1 cm after fixation, neither developed a local recurrence, although one developed a distant recurrence during follow-up.

**Conclusion:**

Formalin fixation results in a measurable reduction in the distal margin length in rectal cancer specimens. Although this reduction is generally modest, it may occasionally influence the pathological interpretation of distal clearance, particularly in borderline cases. These findings should be interpreted in the context of specimen processing and do not necessarily indicate a true oncological inadequacy. Larger studies evaluating long-term oncological outcomes are required to further clarify the clinical implications of fixation-related margin shortening.

## Introduction

The distal resection margin (DRM) is a critical determinant of local control during rectal cancer surgery. Traditional recommendations favored margins of up to 5 cm to minimize recurrence; however, the introduction of total mesorectal excision and the widespread use of neoadjuvant chemoradiotherapy have allowed the use of narrower margins without compromising oncological safety (1). Recent evidence indicates that even subcentimeter margins may be adequate, especially after neoadjuvant treatment (2).

Current literature reflects on the ongoing debate regarding the minimal safe DRM. Some analyses have shown that the DRM length itself is not an independent predictor of survival, while circumferential margin involvement and R1 resection status remain the strongest determinants of outcome (3). Several studies have nevertheless suggested that a margin of ≤1 cm does not negatively affect survival in mid or low rectal cancer. In highly responsive tumors, margins as short as 1 mm may be sufficient, whereas poor responders require a wider clearance margin (4, 5). Earlier reviews have also supported the safety of ≤1 cm in selected patients after neoadjuvant therapy (6). In contrast, other reports cautioned that margins <1 cm can increase recurrence in ypT2–4 disease, highlighting the role of pathological stage (7). Favorable outcomes have also been documented with margins <1 cm, although some analyses emphasize the lack of consensus and recommend post-treatment distances of less than 2 cm (8, 9). Additional evidence suggests that a 1 cm distal margin is oncologically safe in proctectomy, reinforcing the view that shorter margins can be acceptable in selected patients (10). Pathological series consistently show that distal intramural spread rarely exceeds 5–10 mm (11), and some studies suggest that it may even be negligible after neoadjuvant therapy (12). A recent systematic review confirmed that the distal spread seldom extends beyond 20 mm, providing further support for conservative DRM thresholds (13). However, interpreting these findings is complicated by the fact that surgical margins measured intraoperatively may not correspond to the final pathological report due to specimen shrinkage, a phenomenon that has been increasingly recognized but evaluated in relatively few clinical studies.

In addition to the uncertainty regarding the optimal DRM, the interpretation is further complicated by tissue shrinkage after resection and fixation. Formalin exposure has been shown to cause measurable contraction of colorectal specimens, reducing both distal and circumferential margins (14). This raises the possibility that intraoperative assessments may not correspond precisely to final pathological measurements, creating a potential gap between surgical intent and pathological reporting. The present study was designed to evaluate this discrepancy in a contemporary series of rectal cancer patients and further characterize fixation-related changes in distal margin measurements and their potential clinical relevance.

## Methods

### Study design

Between 2019 and 2023, a retrospective observational study was conducted in the Department of General Surgery, Aydın Adnan Menderes University. Patients who underwent anterior resection or low anterior resection for rectal cancer during this period were eligible for inclusion. Only patients with complete clinical and pathological data, including both intraoperative fresh macroscopic distal margin measurement and definitive pathological assessments, were analyzed.

### Inclusion and exclusion criteria

Inclusion criteria included patients with histologically confirmed rectal adenocarcinoma who underwent curative resection through either anterior resection or low anterior resection. Anterior resection was performed for tumors located in the upper rectum (10–15 cm from the anal verge), whereas low anterior resection was chosen for tumors of the mid (5–10 cm) and lower rectum (0–5 cm), in accordance with standard definitions (15). Only patients in whom the distal margin was assessed intraoperatively by fresh macroscopic distal margin measurement (BF: before fixation) and subsequently reevaluated on the formalin-fixed specimen (AF: after fixation) were included. Exclusion criteria included emergency resections, palliative procedures without distal margin assessment, and cases with incomplete clinicopathological data.

### DRM measurements and fixation procedure

During surgery, the distal resection margin was identified and inked on the fresh specimen. Immediately after the fresh specimen arrived at the pathology unit for intraoperative distal margin assessment, the rectal specimen was opened longitudinally along the antimesenteric (anterior) aspect to expose the tumor and distal resection line. The specimen was placed flat on a rigid surface without stretching or manual traction, and the shortest macroscopic distance between the distal tumor edge and the distal resection line was measured using a sterile ruler. This measurement was recorded as the prefixation distance (BF). All fresh measurements were performed by the same pathologist using a standardized institutional protocol. The specimen orientation was maintained throughout subsequent handling.

Following fresh measurements, the specimen was pinned onto a board in an anatomically oriented position without intentional stretching and fixed in 10% neutral-buffered formalin (3.7%–4.0% formaldehyde) using at least a 10:1 fixative-to-tissue volume ratio. Specimens were kept in formalin for 24–48 h at room temperature before routine pathological processing, in accordance with the standard pathology protocols (16). After formalin fixation, the same pathologist remeasured the distal margin macroscopically using the same anatomical landmarks and measurement method, and the value was recorded as the postfixation distance (AF). The difference between BF and AF was calculated in millimeters, and the percentage change was expressed as the shrinkage rate.

The pathologist was not blinded to fresh measurements because the purpose of the protocol was to compare paired measurements within the same specimen. All consecutive eligible patients with both fresh and postfixation distal margin measurements available in the pathology records were included.

### Statistical analyses

Continuous variables were expressed as mean ± standard deviation or median (interquartile range), as appropriate, and categorical variables were summarized as frequencies and percentages. The normality of continuous variables was assessed using the Shapiro–Wilk test. Paired comparisons between BF and AF measurements were performed using the paired *t*-test for normally distributed data and the Wilcoxon signed-rank test for non-normally distributed data. Between-group comparisons were performed using the independent *t*-test or Mann–Whitney U test, as appropriate. Subgroup analyses according to demographic and clinical variables, including age, sex, surgical procedure (anterior resection vs. low anterior resection), tumor level (upper, mid, or low rectum), and neoadjuvant chemoradiotherapy status, were performed using independent *t*-tests or Mann–Whitney *U* tests, as appropriate. Correlations between fixation time and margin change were assessed using Pearson's correlation analysis. All statistical analyses were performed using SPSS software (version 29, IBM Corp., Armonk, NY, USA), and a *p* value <0.05 was considered statistically significant.

### Ethical approval

This study was conducted in accordance with the ethical standards of the Declaration of Helsinki and its later amendments. Ethical approval was obtained from the Ethics Committee of Aydın Adnan Menderes University (Approval No: 2024/137, Date: 25 July 2024). Written informed consent was obtained from all patients prior to inclusion, and all data were anonymized before the analysis.

## Results

### Patient characteristics

A total of 60 patients were included in the final analysis. The median age was 65.5 years (range, 42–81 years), and 34 patients (56.7%) were men. With regard to surgical procedures, patients underwent either anterior resection or low anterior resection. Neoadjuvant chemoradiotherapy was administered to 34 patients (56.7%), while 26 patients (43.3%) underwent surgery without preoperative treatment. The baseline demographic and clinicopathological characteristics are summarized in Table 1.

**Table 1:** Baseline characteristics of the study population.

Variable	Value
Number of patients	60
Age (mean ± SD) (years)	65.4 ± 8.6
Sex (men), *n* (%)	34 (56.7)
Sex (women), *n* (%)	26 (43.3)
Anterior resection, *n* (%)	10 (16.7)
Low anterior resection, *n* (%)	50 (83.3)
Neoadjuvant chemoradiotherapy, *n* (%)	34 (56.7)
No neoadjuvant therapy, *n* (%)	26 (43.3)
Tumor level—upper rectum, *n* (%)	9 (15.0)
Tumor level—mid rectum, *n* (%)	26 (43.3)
Tumor level—low rectum, *n* (%)	25 (41.7)
Fixation time (mean ± SD) (h)	47.7 ± 5.0

### Distal margin measurements before and after fixation

**Figure 1:**
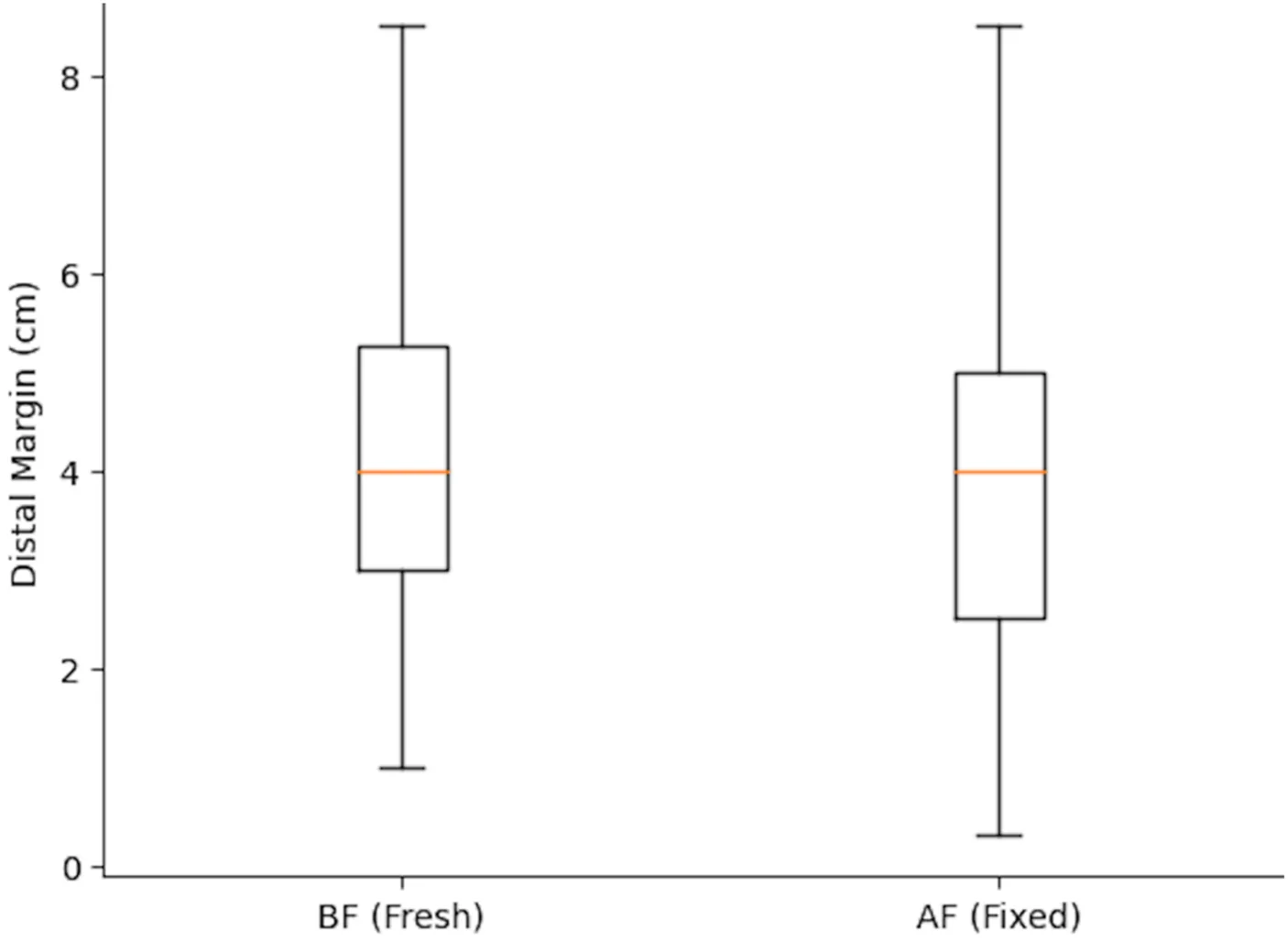
Distribution of distal resection margin measurements before fixation (BF) and after formalin fixation (AF). The box plots illustrate the median, interquartile range, and overall distribution of distal margin lengths in the study cohort. A reduction in the distal margin length after fixation is evident.

**Figure 2:**
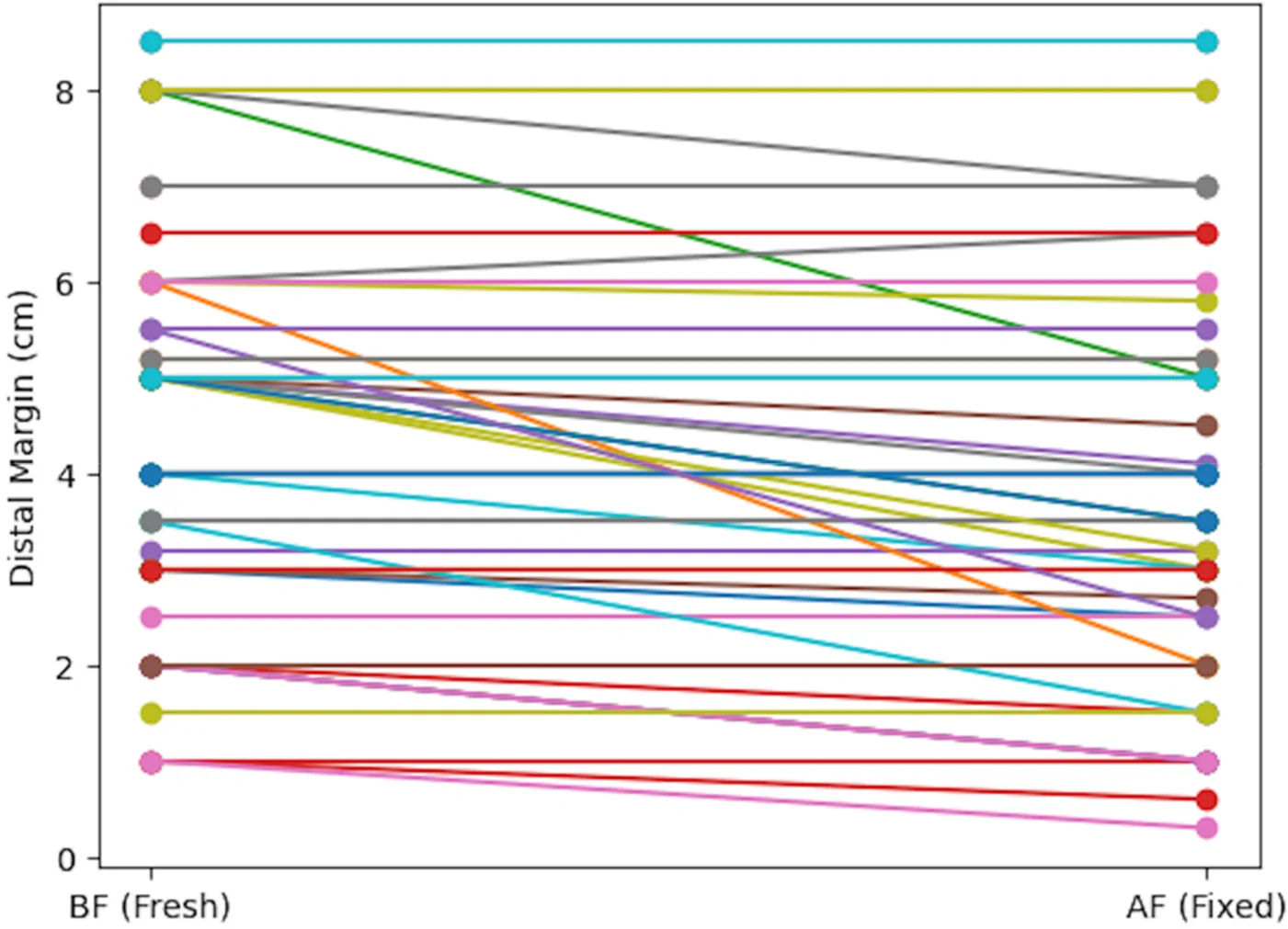
Paired comparison of distal resection margin measurements before fixation (BF) and after formalin fixation (AF). Each line represents an individual specimen. Most paired measurements demonstrate a reduction in the distal margin length following fixation, illustrating the consistent shrinkage effect observed across the cohort.

The mean distal margin measured on the fresh specimen (BF) was 4.39 ± 1.95 cm, whereas the mean distal margin after formalin fixation (AF) was 3.90 ± 2.04 cm. Comparison of paired measurements demonstrated a consistent reduction in the distal margin length following fixation. This decrease in the distal margin length after fixation is illustrated in the box plot comparison (Figure 1). Individual paired comparisons further demonstrated that most specimens showed a reduction in the distal margin length following fixation (Figure 2).

The mean absolute difference between BF and AF measurements was 0.49 cm (95% CI: 0.26–0.71 cm), corresponding to a mean shrinkage rate of approximately 13.2%.

Paired comparison of BF and AF values demonstrated a statistically significant decrease in the distal margin length after formalin fixation (Wilcoxon signed-rank test, *p* < 0.001).

### Distribution of margin changes

**Figure 3:**
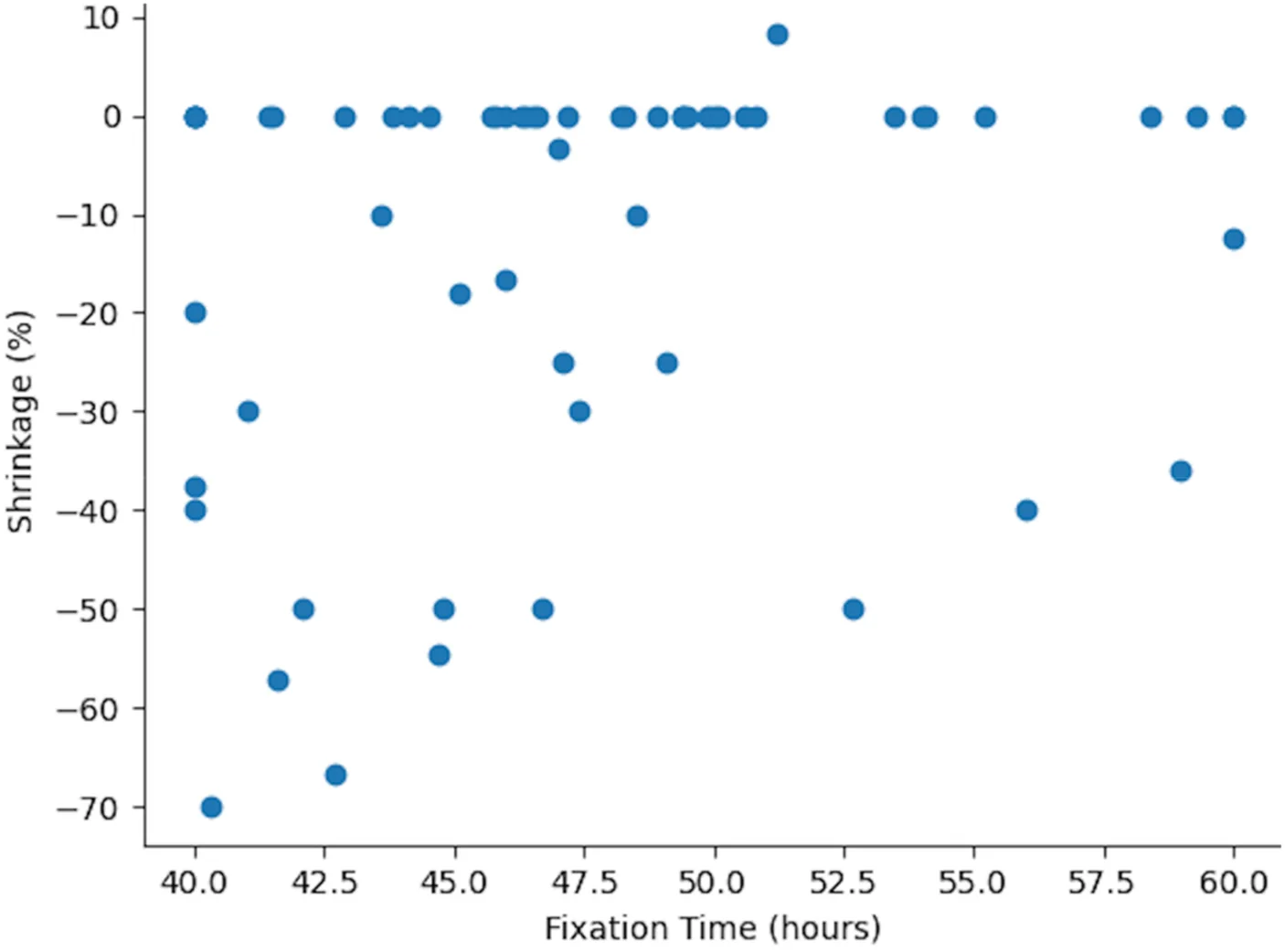
Relationship between fixation duration and percentage shrinkage of the distal resection margin. Each point represents an individual specimen. A weak positive correlation was observed between fixation duration and shrinkage percentage (Pearson's *r* = 0.26, *p* = 0.044), although the effect size was small.

Although most specimens demonstrated a reduction in the distal margin length after fixation, the degree of change varied between cases. The distribution of margin differences and percentage shrinkage across the cohort is presented in Table 2. Correlation analysis demonstrated a weak positive association between the fixation time and shrinkage percentage (Pearson's *r* = 0.26, *p* = 0.044). However, the magnitude of the correlation was small, indicating that the fixation time explained only a limited proportion of the variability in shrinkage. The relationship between the fixation time and shrinkage percentage is presented in Figure 3.

**Table 2:** Comparison of distal resection margin measurements BF and AF.

Measurement	Mean ± SD
Fresh distal margin (BF) (cm)	4.39 ± 1.95
Fixed distal margin (AF) (cm)	3.90 ± 2.04
Mean change (cm)	0.49
Mean shrinkage (%)	13.23

BF, before fixation; AF, after fixation. Mean change was calculated as BF minus AF.

### Subgroup analyses

Subgroup analyses were performed according to sex, neoadjuvant chemoradiotherapy status, and the surgical procedure. No statistically significant differences in shrinkage rates were observed between patients who received neoadjuvant therapy and those who did not (*p* > 0.05). No clear subgroup-specific pattern was detected; however, these exploratory analyses were not powered to detect clinically meaningful subgroup differences and should, therefore, be interpreted with caution.

During a median follow-up of 46.5 months, local recurrence was observed in 13 patients and overall recurrence in 19 patients (Table 3). Two patients had a distal margin of <1 cm after fixation. Neither developed local recurrence during follow-up; however, one patient developed distant recurrence 8 months after surgery, whereas the other remained free of recurrence during the 63 months of follow-up. The margin measurements and oncological outcomes of the two patients are summarized in Table 4. Oncologic outcomes according to the distal margin after fixation are summarized in Table 5. No clear differences in recurrence or mortality patterns were observed between patients with distal margins below and above 2 cm.

**Table 3:** Overall oncologic follow-up outcomes of the study cohort.

Variable	Value
Median follow-up (months)	46.5
Local recurrence, *n* (%)	13 (21.7)
Overall recurrence, *n* (%)	19 (31.7)
Death, *n* (%)	14 (23.3)

**Table 4:** Margin measurements and oncological outcomes of the two patients whose distal resection margins crossed the 1-cm threshold after formalin fixation.

Variable	Patient 1	Patient 2
Fresh distal margin (BF) (cm)	1.0	1.0
Fixed distal margin (AF) (cm)	0.6	0.3
Local recurrence	No	No
Overall recurrence	Yes (8 months)	No
Follow-up (months)	17	63
Survival status	Deceased	Alive

**Table 5:** Exploratory oncologic outcomes according to the distal resection margin measured AF.

Variable	AF <2 cm (*n* = 10)	AF ≥2 cm (*n* = 50)
Local recurrence, *n* (%)	2 (20.0)	11 (22.0)
Overall recurrence, *n* (%)	2 (20.0)	17 (34.0)
Death, *n* (%)	2 (20.0)	12 (24.0)
Median follow-up (months)	46.5	47

Statistical comparisons were not performed because of the limited sample size and the exploratory nature of the analysis. AF represents the distal resection margin measured after fixation.

## Discussion

In the present study, we observed a consistent reduction in the distal resection margin length after formalin fixation compared with the measurements obtained on the fresh specimen. On average, the distal margin decreased by approximately 13%. Although this degree of contraction was modest, it was sufficient in a few cases to change the categorical interpretation of the margin. Notably, none of the specimens had a distal margin shorter than 1 cm in the fresh measurement, whereas two specimens had a margin below this threshold after fixation. Exploratory subgroup analyses did not reveal any clear subgroup-specific patterns based on surgical technique, sex, age, or neoadjuvant chemoradiotherapy. However, these analyses were based on relatively small subgroups and were not powered to exclude clinically meaningful differences. These findings highlight that fixation-related contraction is a relevant factor in all rectal cancer resections and must be considered when interpreting pathological margins.

Previous studies have reported considerably higher rates of shrinkage than those observed in this study. Goldstein described an overall reduction exceeding 50%, most of which occurred shortly after resection and continued until fixation. Ono also reported substantial contraction after formalin exposure, although the magnitude of shrinkage varied considerably depending on experimental conditions (16, 17). Shrinkage of approximately 20% has been documented in colonic specimens, further confirming the impact of specimen handling and fixation time (18). In contrast, we observed an average of 13%, which was lower than that historically reported. This difference may be related to variations in surgical practice, handling, or measurement techniques. Other studies have noted reductions of up to 40%, emphasizing the heterogeneity of this phenomenon (19).

Although the primary aim of this study was not to evaluate oncological outcomes, an additional follow-up analysis was performed to explore the potential clinical relevance of fixation-related shortening of the distal margin. During follow-up, no apparent association in recurrence patterns was observed between patients with distal margins below and above 2 cm after fixation. Moreover, neither of the two patients with a distal margin of <1 cm after fixation developed local recurrence. One patient developed distant recurrence 8 months after surgery, whereas the other remained disease-free throughout the follow-up period. These findings suggest that fixation-related shortening may not necessarily reflect true oncological inadequacy, particularly in borderline cases, as recent evidence indicates that selected patients with short distal margins may still achieve favorable oncological outcomes (20). Nevertheless, given the limited number of patients with short distal margins, these observations should be interpreted cautiously and considered hypothesis-generating, rather than definitive. More recent reports have described shrinkage rates comparable to or somewhat higher than those in our cohort. Pediatric colorectal series documented an average contraction of approximately 23% (21), while an *in vivo*–*in vitro* comparison showed a 25.5% reduction (22). Other studies have found reductions of 35%–40%, although these changes did not increase the risk of local recurrence (23). A similar reduction of approximately 15% was reported elsewhere, where correction factors for fixation-related changes were suggested. Radiotherapy may also modify intramural spread, with proposed correction factors of up to 1.75 in post-treatment specimens (24).

Methodological differences may also explain the variability in the reported shrinkage. Imaging-based assessments have confirmed a reduction of approximately 30% in prospective MRI studies (25). Fixation has also been shown to alter pelvic sidewall lymph node dimensions, suggesting that the effect extends beyond the rectal specimen (26). Beyond imaging, technical aspects such as the timing of measurement, whether the specimen is stretched, and the precision of pathological assessment can all influence the results. These factors highlight the need for standardized measurement protocols across different centers. In the present study, measurements were performed immediately on fresh specimens and repeated after fixation by the same pathologist using a standardized protocol, which may partly explain the relatively modest degree of shrinkage observed.

When comparing our findings with previous reports, differences may arise not only from patient selection but also from handling techniques. The degree of traction applied, the orientation of the specimen, and the formalin exposure time are all important. In the present study, fixation time showed a weak positive correlation with the shrinkage percentage (Pearson's *r* = 0.26, *p* = 0.044). However, the effect size was small, and the relatively narrow range of fixation times limited the clinical significance of this finding, further supporting the need for standardized fixation protocols (27).

Although our analysis highlights fixation-related changes in the distal margins, the clinical implications of this phenomenon remain incompletely understood. In the present cohort, exploratory follow-up analysis did not reveal any obvious association between fixation-related margin shortening and recurrence patterns. Nevertheless, awareness of this discrepancy remains clinically relevant, as even a modest contraction can reduce a 2 cm intraoperative margin to less than 2 cm on the final pathology report. Therefore, surgeons should consider this effect, particularly in low anterior resections, where distal clearance is limited. It should be emphasized, however, that fixation-related shrinkage represents a measurement phenomenon rather than a biological change in tumor extent. All patients in the present study underwent margin-negative resection, and the observed shortening reflected tissue contraction after specimen handling rather than a reduction in true oncological clearance. Therefore, pathological margin measurements should be interpreted in the context of both surgical findings and specimen processing. At the same time, it is important to acknowledge that oncological outcomes are primarily determined by tumor biology and overall margin status rather than by small fixation-related changes in measured margin length. Circumferential margin involvement remains one of the strongest prognostic factors and continues to guide oncological risk assessment in rectal cancer (28).

### Limitations

This study has several limitations. First, it was a retrospective single-center analysis with a relatively limited sample size, which may restrict the generalizability of the findings. Second, only patients with both fresh and postfixation distal margin measurements available in the pathology records were included, which may have introduced a selection bias. Third, although additional oncological follow-up data were evaluated, the number of patients with very short distal margins was limited; therefore, the oncological analyses should be interpreted cautiously. Finally, although measurements were performed using a standardized protocol, minor variations in specimen orientation, tissue elasticity, and fixation conditions may still have influenced the recorded distances. Nevertheless, all measurements were performed by the same pathologist using a consistent methodology, which improved the internal consistency of the measurements, although a formal assessment of measurement reproducibility was not performed. In addition, the exploratory oncological analyses were based on a limited number of patients with short distal margins and should not be interpreted as establishing a universal correction factor or a practice-changing distal margin threshold. External validation in larger prospective cohorts is required.

## Conclusion

Formalin fixation results in a measurable reduction in the distal resection margin length in rectal cancer specimens. In our study, the distal margin decreased by approximately 13% on average when measurements obtained from fresh specimens were compared with those recorded after the fixation. Although this reduction is generally modest, it may occasionally alter the categorical interpretation of distal clearance in some cases. Surgeons and pathologists should, therefore, be aware that the distal margin reported in the final pathology assessment may be shorter than the intraoperative measurement obtained on fresh specimens. Further studies evaluating larger cohorts and long-term oncological outcomes are required.

## Data Availability

The data analyzed in this study are subject to the following licenses/restrictions: The dataset contains deidentified clinical data from human participants and cannot be made publicly available because of institutional ethics and patient confidentiality requirements. Deidentified data are available from the corresponding author upon reasonable request and with approval from the Institutional Ethics Committee, where applicable. Requests for access to these datasets should be directed to Akay Edizsoy, akayedizsoy@adu.edu.tr.

## References

[1] Manegold P Taukert J Neeff H Fichtner-Feigl S Thomusch O. The minimum distal resection margin in rectal cancer surgery and its impact on local recurrence—a retrospective cohort analysis. Int J Surg. (2019) 69:77–83. 10.1016/j.ijsu.2019.07.029 31362126

[2] Zhang C Cui M Xing J Yang H Su X. Oncological results in rectal cancer patients with a subcentimetre distal margin after laparoscopic-assisted sphincter-preserving surgery. ANZ J Surg. (2022) 92(6):1454–60. 10.1111/ans.17503 35088533 PMC9305552

[3] Carbone F Santalucia R Borin S Ciardiello D Bottiglieri L De Pascale S. Impact of distal resection margin on survival after tumour-specific mesorectal excision for rectal cancer: retrospective cohort study. Clin Colorectal Cancer. (2025). 10.1016/j.clcc.2025.08.001 40962615

[4] Yan H Wang PY Wu YC Liu YC. Is a distal resection margin of ≤ 1 cm safe in patients with intermediate- to low-lying rectal cancer? A systematic review and meta-analysis. J Gastrointest Surg. (2022) 26(8):1791–803. 10.1007/s11605-022-05342-9 35501549

[5] Sorrentino L Sileo A Daveri E Battaglia L Guaglio M Centonze G. Impact of microscopically positive (≤1 mm) distal margins on disease recurrence in rectal cancer treated by neoadjuvant chemoradiotherapy. Cancers (Basel). (2023) 15(6):1828. 10.3390/cancers15061828 36980714 PMC10047023

[6] Bujko K Rutkowski A Chang GJ Michalski W Chmielik E Kusnierz J. Is the 1-cm rule of distal bowel resection margin in rectal cancer based on clinical evidence? A systematic review. Ann Surg Oncol. (2012) 19(3):801–8. 10.1245/s10434-011-2035-2 21879269 PMC3278608

[7] Song SH Park JS Choi GS Seo AN Park SY Kim HJ. Impact of the distal resection margin on local recurrence after neoadjuvant chemoradiation and rectal excision for locally advanced rectal cancer. Sci Rep. (2021) 11(1):22943. 10.1038/s41598-021-02438-1 34824330 PMC8617265

[8] Yu G Chi H Zhao G Wang Y. Tumor regression and safe distance of distal margin after neoadjuvant therapy for rectal cancer. Front Oncol. (2024) 14:1375334. 10.3389/fonc.2024.1375334 38638858 PMC11024319

[9] Luo J Zhu M Zhao L He M Li B Liu Y. Distal margin distance in radical resection of locally advanced rectal cancer after neoadjuvant therapy. Chin J Cancer Res. (2024) 36(2):226–32. 10.21147/j.issn.1000-9604.2024.02.09 38751434 PMC11090793

[10] Li M Wang X Niu P Yang Y Chen J Xu J. Prognostic implications of distal resection margin length in rectal cancer proctectomy: a retrospective analysis. J Clin Oncol. (2024) 42:e15605. 10.1200/JCO.2024.42.16_suppl.e15605

[11] Lin S Wei J Lai H Zhu Y Gong H Wei C. Determining the optimal distal resection margin in rectal cancer patients by imaging of large pathological sections: an experimental study. Medicine (Baltimore). (2024) 103(21):e38083. 10.1097/MD.0000000000038083 38787988 PMC11124751

[12] Guedj N Maggiori L Poté N Norkowski E Cros J Bedossa P. Distal intramural and tumor spread in the mesorectum after neoadjuvant radiochemotherapy in rectal cancer: about 124 consecutive patients. Hum Pathol. (2016) 52:164–72. 10.1016/j.humpath.2016.01.017 27210028

[13] Grüter AAJ Van Lieshout AS Van Oostendorp SE Ket JCF Tenhagen M Den Boer FC. Required distal mesorectal resection margin in partial mesorectal excision: a systematic review on distal mesorectal spread. Tech Coloproctol. (2023) 27(1):11–21. 10.1007/s10151-022-02690-1 36036328 PMC9807492

[14] Lam D Kaneko Y Scarlett A D'Souza B Norris R Woods R. The effect of formalin fixation on resection margins in colorectal cancer. Int J Surg Pathol. (2019) 27(7):700–5. 10.1177/1066896919854159 31195869

[15] Glimelius B Tiret E Cervantes A Arnold D, ESMO Guidelines Working Group. Rectal cancer: ESMO clinical practice guidelines for diagnosis, treatment and follow-up. Ann Oncol. (2013) 24(Suppl 6):vi81–8. 10.1093/annonc/mdt240 24078665

[16] Goldstein NS Soman A Sacksner J. Disparate surgical margin lengths of colorectal resection specimens between *in vivo* and *in vitro* measurements. The effects of surgical resection and formalin fixation on organ shrinkage. Am J Clin Pathol. (1999) 111(3):349–51. 10.1093/ajcp/111.3.349 10078110

[17] Ono C Yoshinaga K Enomoto M Sugihara K. Discontinuous rectal cancer spread in the mesorectum and the optimal distal clearance margin in situ. Dis Colon Rectum. (2002) 45(6):744–9; discussion 742–3. 10.1007/s10350-004-6290-1 12072624

[18] Ho YM Hoff J May A Renwick C. Distal margin shrinkage factor—a consideration before dividing the specimen in colorectal cancer surgery. Iran J Colorectal Res. (2020) 8(2):75–8. 10.30476/ACRR.2020.46702

[19] Garg P Kumar N. Effect on surgical margins following formalin fixation. J Popul Ther Clin Pharmacol. (2025) 32(5):1306–10. 10.53555/rhs3fc03

[20] Lau SYC Lu CT Lam AK. Changes in surgical margins following formalin fixation and the incidence of local recurrence of colorectal cancer. Int Surg J. (2025) 12(4):508–11. 10.18203/2349-2902.isj20250806

[21] Gretser S Weber KJ Braun Y Harter PN Rolle U McNally J. Tissue shrinkage of resected specimens in Hirschsprung's disease: why pediatric surgeons think the bowel specimen was longer than indicated in the pathology report. Pediatr Dev Pathol. (2023) 26(3):287–91. 10.1177/10935266231162684 36994845 PMC10291109

[22] Gorbunova AS Aniskin AA Kuzmichev DV Mamedli ZZ Полыновский АВ Lovenger AA. Distal resection margin in colorectal surgery *in vivo* and after formalin fixation. Surg. Oncol. (2025) 15:36–41. 10.17650/2949-5857-2025-15-1-36-41

[23] Aday U Kılıçarslan A Böyük A Akkoç H. Investigation into the effect of neoadjuvant therapy and tumor regression grade on the shrinkage of distal surgical margin in rectal cancer: a prospective case-control study. Indian J Pathol Microbiol. (2022) 65(2):343–8. 10.4103/IJPM.IJPM_1130_20 35435369

[24] Sun G Ye X Zheng K Zhang H Broens P Trzpis M. Measurement of distal intramural spread and the optimal distal resection by naked eyes after neoadjuvant radiation for rectal cancers. World J Surg Oncol. (2022) 20(1):296. 10.1186/s12957-022-02756-2 36104818 PMC9472430

[25] Bondeven P Hagemann-Madsen RH Bro L Moran BJ Laurberg S Pedersen BG. Objective measurement of the distal resection margin by MRI of the fresh and fixed specimen after partial mesorectal excision for rectal cancer: 5 cm is not just 5 cm and depends on when measured. Acta Radiol. (2016) 57(7):789–95. 10.1177/0284185115604007 26377262

[26] Kawai K Morikawa T. The effect of formalin fixation on the size of pelvic sidewall lymph nodes. Int J Colorectal Dis. (2018) 33(10):1493–5. 10.1007/s00384-018-3103-x 29926234

[27] Horn CL Naugler C. Breast specimen shrinkage following formalin fixation. Pathol Lab Med Int. (2014) 6:11–4. 10.2147/PLMI.S59842

[28] Alipouriani A Almadi F Rosen DR Liska D Kanters AE Ban K. Margin matters: analyzing the impact of circumferential margin involvement on survival and recurrence after incomplete total mesorectal excision for rectal cancer. Tech Coloproctol. (2025) 29(1):50. 10.1007/s10151-024-03098-9 39847185 PMC11757853

